# Supplementary material for: Learning curve and surgical outcome of robotic assisted colorectal surgery with ERAS program
Source: Sci Rep. 2022 Nov 29;12:20566. doi: 10.1038/s41598-022-24665-w (PMC9709162; doi:10.1038/s41598-022-24665-w)
Supplement: Supplementary file 2 — Supplementary Information 2. [file 41598_2022_24665_MOESM2_ESM.zip › RAL Raw data-Ñ[▒K/141/OP N_ñw╝╨░O▒Kñσ.pdf]

W52 / W52

臺中榮民總醫院
手術室護理記錄

[redacted]

基本資料

- 室別： 開刀房-17室，手術科別： CRS，手術類別： 常規，口期： 2022/01/07
- 進入時間： 1405 ； 麻醉開始時間： 1415 ； Time Out時間： 1540 ； 劃刀時間： 1541 ； 手術結束時間： 2045 送出時間： 2110 ；
- 麻醉方式： GE

  診斷： Malignant tumor of rectum 8CM

  手術名稱： Robot Assisted Low Anterior Resection

  手術局部用藥： NIL

  術前預防性抗生素：
  - 抗生素名稱一： Veterin 1GM IVD 由麻醉護理師給予
  - 時間： 1525
  - 上次簽章: 邱儷嫀

  ，追加預防性抗生素：
  - 抗生素名稱一： Veterin 1GM IVD 由麻醉護理師給予
  - 時間： 1930
  - 記錄者： 許臺真
- 手術醫師： 蔣鋒帆，楊曙榮，陳瑋夫，林志安，陳凱祥，陳威遠
- 麻醉醫師： VS 陳永銘
- 刷手護士A ： 何采穎 ，時間： 1405 ，接替刷手一： 邱任宏 ，時間： 1500 ，接替刷手二： 邱儷嫀 ，時間： 1730

  流動護士A ： 黃宸盈 ，時間： 1405 ，流動護士B ： 歐真勤 ，時間： 1405 ，接替流動一： 許臺真 ，時間： 1500，接替流動二： 邱儷嫀，時間： 1500
- 更多接替及流動護士： 接替流動三： 許臺真，時間： 1700

皮膚狀況

A-V Shunt： 無

術前： 不完整

部位(術前)：

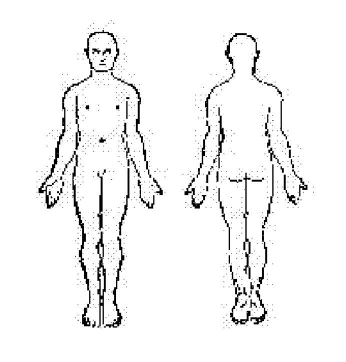

左膝蓋破皮

記錄者： 上次簽章: 黃宸盈

術後： 不完整

部位(術後)：

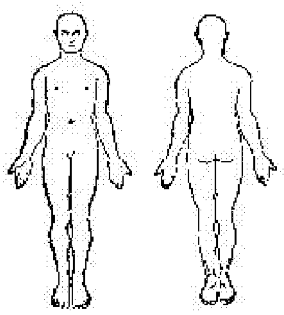

同術前
術後左髖15x3cm壓紅不可白

記錄者： 上次簽章: 邱欐婇

手術準備

手術臥式 單- -手術臥式

-
  - 手術臥式： 截石臥式
  - 使用附件： 腳架，其它： 海棉盒
  - 皮膚準備： 10%Aq Betadine，Hibiscrub，2% Chlorhexidine
  - 電燒： 使用電燒: 電極片位置： 其它： 迴路電極墊，記錄者： 上次簽章: 黃宸盈

止血帶T： 無

植入物

- da vinci xi endowrist stapler 45-4.3mm*2/rectum
  surgiwrap 100*130mm/腹

, 記錄者: 上次簽章: 許臺真

標本送檢

- 細菌培養 無
- 細胞學 無
- 冰凍切片 無
- 病理標本
  組織名稱一：rectum
  ,
  組織名稱二：distal cut end
  ,
  組織名稱三：IMA Root
- 截肢 無

標本交付 標本： 1 袋，標本： 0 盒，標本： 2 瓶，細胞學： 0 管，Culture Tube： 0 管，被交付醫師： CR 陳璿夫，

- 記錄者： 上次簽章: 許臺真
- 時間： 1900

傷口術後敷料

傷口敷料： 傷口敷料： 無

- 請於圖上標示部位：

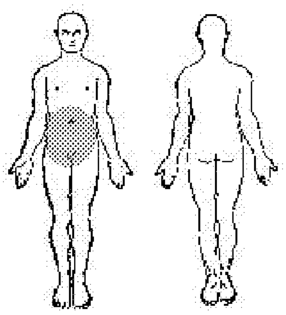

-

- 傷口敷料： 傷口敷料

導管

導管	天數	導管資訊	插入日期	插入時間	更換日期	拔除日期	拔除時間	拔除原因	預計拔除/更換	預計拔除/更換日期
Double Lumen CVC(1)	1	號碼:8 Fr; 深度:15cm; 部位:subclavian (右); 象限:RU;	2022/01/07	1500						
A-line (1)	1	號碼:20 G; 部位:左手;	2022/01/07	1450						
Foley(1)	1	號碼:16 Fr.; 部位:膀胱;	2022/01/07	1430						
Epidural PCA(1)	1	部位:硬脊膜;	2022/01/07	1430						

用物計數

- 手術中出血量： minimal c.c.
- 手術中尿量： 250 c.c.
-
    - 其它： 無

用物計數： 多切口同時縫合

- 1.器械： 正確，2.縫針： 正確，3.刀片： 正確，4.紗布： 正確，5.腹部墊： 未使用，6.紗球： 未使用，7.PEANUTS ： 未使用，8.紗條： 未使用，9.絲帶： 未使用，10.針頭： 未使用，11.Vessel loop： 未使用，12.Boots： 未使用，13.Jelco： 未使用，14.棉條： 未使用，
    - 第一次：
        - 刷手護士 ： 邱權婇
        - 流動護士： 上次簽章: 許臺真
        - 時間： 1910

  ,
    - 第二次：
        - 刷手護士 ： 邱權婇
        - 流動護士： 上次簽章: 許臺真
        - 時間： 1920

  ,
    - 第三次：
        - 刷手護士 ： 邱權婇
        - 流動護士： 上次簽章: 許臺真
        - 時間： 1930

備註

備註： R17 30度SCOPY NO1

- 記錄者： 上次簽章: 黃宸盈
- 時間： 1430

填寫護理人員 許臺真

正式摘要

W52 / W52

doctorRank:B
